# Concomitant Use of Hyaluronic Acid and Laser in Facial Rejuvenation

**DOI:** 10.1007/s00266-019-01393-7

**Published:** 2019-05-09

**Authors:** Fernando Urdiales-Gálvez, Sandra Martín-Sánchez, Mónica Maíz-Jiménez, Antonio Castellano-Miralla, Leonardo Lionetti-Leone

**Affiliations:** Instituto Médico Miramar, Paseo de Miramar 21, 29016 Málaga, Spain

**Keywords:** Laser, Hyaluronic acid, Dermal fillers, Aesthetics, Intense pulsed light

## Abstract

**Background:**

Facial aging is a process that involves many different changes. Therefore, in many patients, it may be necessary to perform a combined treatment. Botulinum toxin A and dermal fillers are the two most popular nonsurgical cosmetic procedures performed globally to treat age-associated changes. However, there are not many studies reporting the concomitant use of dermal fillers and laser technology for facial rejuvenation. This review aims to assess the concomitant use of dermal hyaluronic acid (HA) fillers and laser technology for facial rejuvenation.

**Methods:**

The present updated consensus recommendations are based on the experience and opinions of the authors and on a literature search.

**Results:**

If a combined procedure (HA and light treatments) is to be performed, on the same day, the panel recommends starting always with the light treatments, avoiding skin manipulations after having injected HA. To customize the therapeutic management, it is crucial to establish a precise diagnosis of the photodamage and loss of volumes suffered by the patients.

**Conclusions:**

The currently available scientific evidence about the combined use of HA fillers and laser–radiofrequency–intense pulsed light (laser/RF/IPL) is limited and encompasses mainly small and nonrandomized studies. Nevertheless, most of these studies found that, on average, the concomitant use (same day) of laser and HA fillers for facial rejuvenation represents an effective and safe strategy which improves clinical results and patient’s satisfaction. Future well-designed clinical studies are needed regarding the effectiveness and safety of combination filler/laser treatments.

**Level of Evidence IV:**

This journal requires that authors assign a level of evidence to each article. For a full description of these Evidence-Based Medicine ratings, please refer to the Table of Contents or the online Instructions to Authors www.springer.com/00266.

## Introduction

The facial aging process is a multifactorial, complex, three-dimensional (3D), dynamic, and generally not uniform process with anatomical, biochemical, and genetic correlates [1–3]. All people age differently as a result of imbalance, disharmony, and disproportion of the aging process between the overlying soft tissue and the underlying bony frameworks.

Aging is a result of the interplay of changes occurring in all five facial anatomical layers: skeleton, ligaments, muscles, adipose tissue, and skin. To target these, multilayer, combined intervention is required to relax, volumize, resurface and re-drape facial skin [4].

Facial aging is associated with loss of soft tissue fullness in certain areas (periorbital, forehead, malar, temporal, mandibular, mental, glabellar and perioral sites) and persistence or hypertrophy of fat in others (submental, lateral nasolabial fold and labiomental crease, jowls, infraorbital fat pouches and malar fat pad) [1, 5].

Facial skin aging is caused by intrinsic and extrinsic mechanisms. Various studies showed that different exogenous and endogenous factors such as solar exposure [6, 7], cigarette smoking [6–8], medications [7], alcohol use [7], gravity [9], body mass index [6], work status [1], mental stress [1], diet [1] and endocrinology status [10] may affect face appearance during aging.

Because the facial aging process involves many different changes, in many patients it may be necessary to perform a combined treatment. The key question is when and how to combine safely and effectively different aesthetic interventions for the face, hands, neck, and décolletage [4, 11, 12].

Optimal outcomes are dependent upon choosing the appropriate tool and ensuring that it is used correctly. A deep understanding of product characteristics, anatomy, and the physiology of aging is essential to know when, where, and how to use different modalities to provide facial harmony.

Two consensus recommendations for the optimal combination and ideal sequence of botulinum toxin A (BoNTA), hyaluronic acid (HA), calcium hydroxylapatite and microfocused ultrasound with visualization (MFU-V) in persons of all Fitzpatrick skin types have been recently published [11, 12].

BoNTA and dermal fillers are the two most popular nonsurgical cosmetic procedures performed globally to treat age-associated changes [13]. In fact, the figures from the American Society of Plastic Surgeons indicate that BoNTA and dermal fillers were the two most common nonsurgical aesthetic treatments in 2014, with more than 3.5 and 1.6 million people receiving such interventions, respectively [13].

However, there are not many studies reporting the concomitant use of dermal fillers and laser technology for facial rejuvenation. It was suggested that the use of laser devices after injection of filling substances might substantially reduce the effect of the fillers and/or lead to rapid degradation of the filling substances. Moreover, the combined treatment with a nonablative infrared device and HA filler does not have enhanced efficacy in treating nasolabial fold wrinkles [14].

Nevertheless, other studies have found that laser, radiofrequency (RF), and intense pulsed light (IPL) treatments can safely be administered immediately after HA gel implantation without reduction in overall clinical effect [15, 16]. Moreover, the use of RF before [17] or after [18] HA filler injection may represent a biocompatible and long-lasting advance in skin rejuvenation.

The objective of this review is to assess the concomitant use of dermal fillers and laser technology for face rejuvenation.

## Materials and Methods

The present updated consensus recommendations are based on the experience and opinions of the authors, and on a literature search conducted in PubMed using the search terms “Laser” OR “Dermal Fillers” OR “Hyaluronic acid” OR “Tissue Interaction” OR “Laser indication” OR “Esthetics”. We selected publications that were published in English, French, and Spanish to date. References cited in selected articles were also reviewed to identify additional relevant reports. Additionally, relevant published national and international guidelines were also scrutinized.

Consensus was achieved by discussion of the evidence and focusing on the scope of the recommendations. An initial document was drafted by the Coordinating Committee, and it was reviewed by the expert panel members. The Coordinating Committee evaluated the panel’s comments and modified the draft as they considered necessary. Subsequent revisions were based on feedback from the other authors until a consensus was achieved, and the final text was then validated.

## Dermal Fillers

Dermal fillers have become very popular over the past few years, and they are mainly used to create a volume or to reverse any loss in the original volume of the face and neck [13]. Derivatives of HA, a natural polysaccharide and a component of the human dermis and epidermis, are probably the biodegradable fillers most widely used in Europe and the USA [13, 19].

Their effect generally lasts 6–18 months depending on the source, the extent of cross-linking and the concentration and particle size of each product [20]. HA products are characterized by the size of their microspheres, and biphasic fillers contain a range of microsphere sizes, such as Restylane^®^ (Medicis Aesthetics, Scottsdale, AZ, USA). Conversely, monophasic HA products like Juvederm^®^ (Allergan, Irvine, CA, USA) contain homogeneous microspheres, that seem to make the gel smoother and more efficient [21, 22].

Different families of monophasic monodensified fillers exist depending on the manufacturing technology, such as the Hylacross^®^ technology (e.g., Juvéderm^®^ Ultra) [23] or the VYCROSS^®^ technology (e.g., Juvéderm^®^ Volbella) [24].

Juvéderm^®^ is derived from *Streptococcus equi* and manufactured by a bacterial fermentation process. Juvéderm^®^ is produced by a proprietary manufacturing process referred to as “Hylacross technology,” which refers to the fact that Juvéderm is not “sized” in contrast to the other HA fillers (Prevelle Silk^®^, Restylane^®^, Perlane^®^) which use sizing technology [23].

Juvéderm^®^ Volbella is a 15-mg/mL HA dermal filler that has been developed using the VYCROSS^®^ technology platform (developed by Allergan Inc., Irvine, CA, USA) and is formulated using a majority of low molecular weight HA together with a minority of high molecular weight HA (> 1 MDa) [24]. This formulation has more efficient cross-linking, which affects the rheology of the product in tissues and the hydrophilic properties of the HA gel. The optimized homogenous matrix is smooth rather than granular; this forms a highly malleable gel that is expected to distribute evenly in the treated tissue [24].

In general, a higher degree of cross-linking makes an HA filler more resistant to enzymatic and free radical degradation, therefore increasing its longevity in the tissues [25].

## Laser and Intense Pulsed Light Therapies

The use of lasers in photoaging began with CO_2_ (10,600 nm). In 1985, the use of this device for the treatment of actinic cheilitis was reported for the first time [26]. In 1989, it was first used for resurfacing of a face with prominent photoaging and multiple actinic cheilitis [27]. In 1991, it was approved by the US Food and Drug Administration for skin renewal, leading to its increased use for actinic keratosis lesions, as well as for the improvement of wrinkles and flaccidity [28–31].

Four major resurfacing laser platforms with dermatologic applications include ablative and nonablative lasers of both the fractionated or nonfractionated types.

Ablative skin resurfacing using the carbon dioxide laser was long considered the gold standard for treatment of photoaging, acne scars, and rhytids [32]. However, conventional full-face carbon dioxide resurfacing is associated with significant risk of side effects and a prolonged postoperative recovery period [32].

The nonablative laser was then developed in the quest of a treatment to improve photoaging with fewer side effects [33–35]. The term “nonablative” was first coined to describe treatment that selectively damages the dermal tissue while sparing the epidermis. In contrast to ablative lasers, nonablative fractional devices are associated with minimal side effects and downtime [36, 37].

The goal of nonablative lasers was to stimulate collagen in the dermis without causing ablation of the epidermis. To this end, 800-nm diode lasers and neodymium-doped yttrium–aluminum–garnet 1064 nm long pulse were used. The results, however, were unsatisfactory, and the procedure did not become as popular as expected [34].

Nevertheless, nonablative laser resurfacing using the 1320-nm neodymium-doped yttrium–aluminum–garnet (Nd:YAG) laser has been shown to produce subtle positive results in patients with minimal downtime and complications [38, 39].

A side-by-side comparison of perioral rhytids treated with an intense pulse light device and the 1064-nm Nd:YAG laser demonstrated similar improvement in rhytid reduction, whereas the 1064-nm Nd:YAG laser was associated with fewer complications and better patient tolerance [40]. Furthermore, the 1064-nm Nd:YAG laser was well tolerated by patients of all skin types [33].

Manstein et al. in 2004 performed a small revolution with the description of the fractionated radiation for the treatment of photoaging [41]. The stimulation of collagen occurred through fractional laser beams, which would reach the selected area while saving islands of sound skin [42].

The nonablative fractional lasers comprise wavelengths of 1440, 1540, 1550 and 1565 nm. Such lengths are well absorbed by water, being a logical choice for the stimulation of collagen remodeling [43].

Fractional resurfacing thermally ablates microscopic columns of epidermal and dermal tissue in regularly spaced arrays over a fraction of the skin surface [44]. This intermediate approach increases the efficacy as compared to nonablative resurfacing, but with faster recovery as compared to ablative resurfacing [44].

There are two commonly used technologies. The erbium glass laser rod (wavelength of 1540 nm) releases rays in a static manner, as is “stamping” the skin. The pulse lasts for 10–100 ms; the fluences used vary from 20 to 100 mJ/cm^2^. On the other hand, the erbium glass laser (wavelength of 1550 nm) releases the rays dynamically, as a “scanner” [42].

The common types of lasers used in aesthetic medicine are summarized in Table 1.

**Table 1:** Common types of lasers used in aesthetic medicine. Adapted from Meaike et al. [45]

Laser name	Wavelength (nm)	Primary chromophore	Indications
Ruby	347	Melanin	Tattoos
Alexandrite	750	Melanin	Tattoos
Intense pulsed light	400–1200	Melanin and hemoglobin	Rosacea, vascular lesions, acne, red tattoos
Nd:YAG	1064, 1320, 1540	Water	Hair removal, deep hemangiomas, black and green tattoos, nevus of Ota
Diode	1450	Water	Hair removal, darker tattoos
Er:YAG	2490	Water	Skin lightening and leveling
CO_2_	10,600	Water	Deep rhytids, sun damage, skin tightening, hypertrophic burn scars

Nd:YAG, neodymium-doped yttrium–aluminum–garnet; Er:YAG, erbium: yttrium–aluminum–garnet

The intense pulsed light (IPL) is a nonlaser-filtered flash lamp device. It is technically not a laser because it is not monochromatic and carries a variety of wavelengths [45]. However, it is treated like a laser, often replacing the pulsed dye laser in many clinical settings [45].

Unlike lasers, IPL devices emit polychromatic, noncoherent and noncollimated light (420–1400 nm) with varying pulse durations [46]. The wider range of light can be absorbed by a variety of chromophores, making IPL less selective than lasers. As such, cutoff filters are often used to narrow the spectrum of emitted wavelengths and render the device more specific [46].

## Laser and IPL Indications

**Fig. 1:**
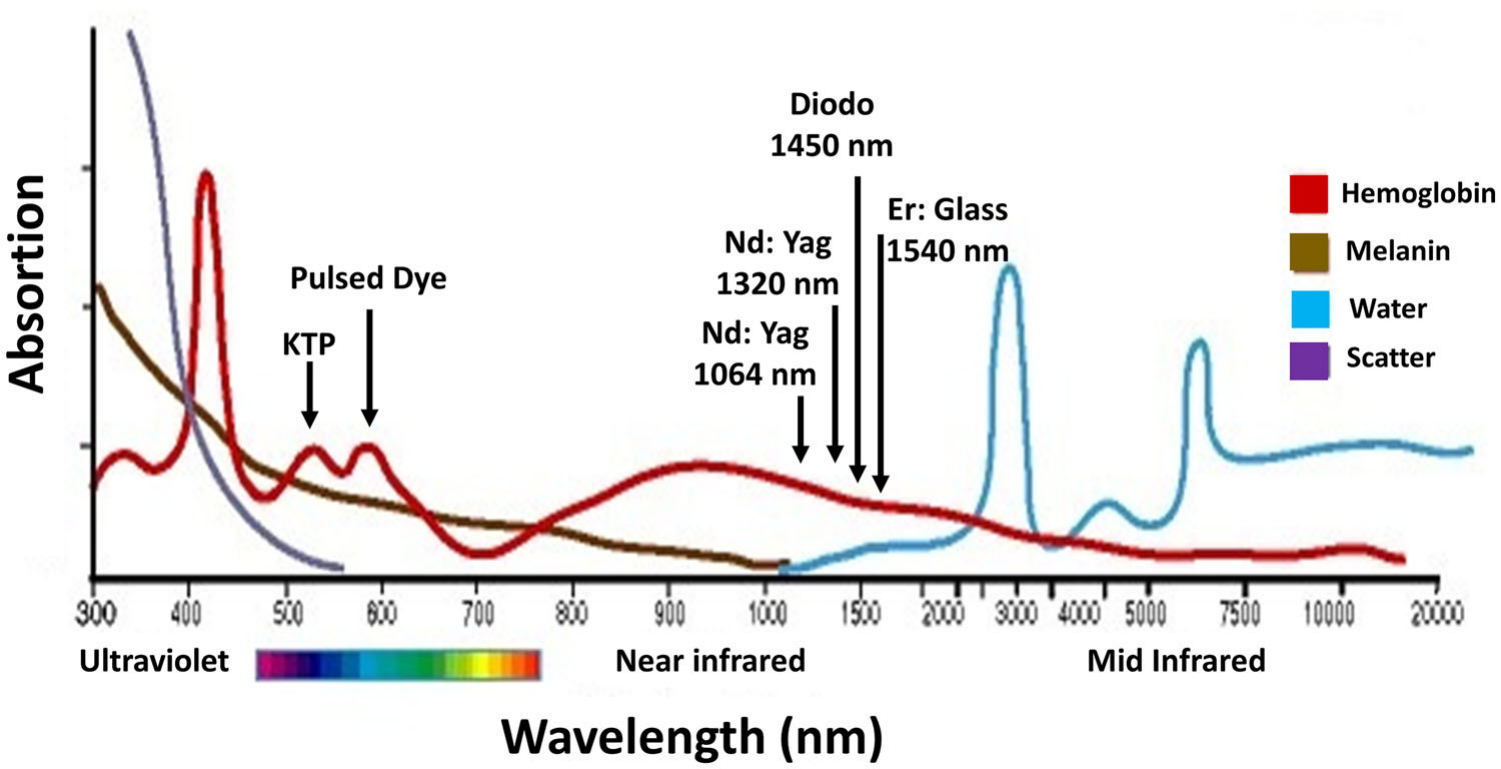
Absorption versus wavelength for various lasers used in aesthetic treatments. Visible light lasers are strongly absorbed by blood (hemoglobin) and pigment (melanin), in contrast to infrared lasers, which are strongly absorbed by water. *KPG* potassium titanyl phosphate,*Nd* neodymium, *YAG* yttrium–aluminum–garnet, *Er* erbium

Lasers can be adjusted to target specific tissues of various cutaneous depths depending upon absorption and scattering profiles of the tissue of interest. The desired effects of lasers are attained when tissues absorb the light energy. Endogenous chromophores (primarily water, melanin and hemoglobin) in the target tissue have wavelength absorption profiles and determine the degree of light absorption (Fig. 1).

### Vascular Lesions

Due to the systems’ ability to specifically target intravascular oxyhemoglobin, vascular lesions are frequently treated with lasers and IPL. This endogenous chromophore has three primary absorption peaks within the visible light spectrum: 418, 542 and 577 nm. Oxyhemoglobin absorbs the laser light, which is subsequently converted to heat and transferred to the vessel wall causing coagulation and vessel closure [46].

Currently, the most commonly used vascular lasers are the potassium titanyl phosphate (KTP, 532 nm), pulsed dye laser (PDL, 585–595 nm), alexandrite (755 nm), diode (800–810, 940 nm), and Nd:YAG (532 and 1064 nm) [46]. In addition, IPL with appropriate filters can be used to treat certain vascular lesions, but the level of recommendation is low [47].

### Hypertrophic Scars, Keloids, and Striae

Hypertrophic scars and keloids are abnormal wound responses to cutaneous injury and are marked by excessive collagen formation. Their therapeutic management is difficult and has high recurrence rates following conventional treatments such as surgical excision, dermabrasion, radiation, and intralesional therapy [48–50].

PDL has been shown to be effective for treating hypertrophic scars, with minimal side effects [51–53].

Striae have been treated successfully with low-fluence PDL, with striae rubra showing greater clinical response to treatment than mature striae alba [54].

### Treatment of Pigmented Lesions

Quality-switched (QS) lasers are highly effective in lightening or eliminating benign epidermal and dermal pigmented lesions [46]. These types of lasers have also been used to treat amateur, professional, and traumatic tattoos [46].

The red and infrared wavelengths of the QS lasers target melanin within melanosomes (as is the case with pigmented lesions) and various carbon-based material or organometallic dyes (as is the case with tattoos), with limited injury to adjacent normal tissue [55].

Although the QS ruby was the first system developed to treat pigmented lesions and tattoos and was widely and successfully used [56, 57], the most recently developed Q-switched lasers have shown an even greater ability to target and destroy cutaneous pigment and ink [58].

## Laser and Tissue Interactions

Hyaluronic acid is a high molecular weight, nonsulfated glycosaminoglycan component that is typically present as a high molecular weight (HMW) biopolymer (MW > 10^6^ Da) in the extracellular matrix of various tissues [59].

It is one of the most hygroscopic molecules in nature, and hydrated hyaluronic acid can contain up to 1000-fold more water than its own weight [60]. These exceptional water retention properties result in enhanced hydration of the skin after the esthetic treatment.

The VYCROSS^®^ technology platform (developed by Allergan Inc., Irvine, CA, USA) has more efficient cross-linking, which affects the rheology of the product in tissues and the hydrophilic properties of the HA gel [24]. VYCROSS allows a total integration in the skin due to its homogeneous matrix structure; this forms a highly malleable gel which evenly distributes in the treated tissue replacing the aging loss of HA [24].

This hydrophilic capacity of HA causes an increase in volume, which is useful for the recovery of facial volumes in the treatment of facial lipoatrophy [23–25].

Approximately 50% of the total quantity of HA in the human body is concentrated in the skin, and it has a half-life of 24–48 h [61]. HA is cross-linked to increase its longevity, and 1,4-butanediol diglycidyl ether is the cross-linking agent used to stabilize the majority of the HA-based dermal fillers currently available on the market [62]. The superior stability of the ether bond (relative to the ester or amide bond) is one of the reasons that BDDE–cross-linked HA fillers have a clinical duration that can reach 12–18 months [62]. These processes enhance the resistance of HA to heat, mechanical stresses, enzymatic degradation and the effect of free radicals [62]. Although the characteristics of the BDDE–cross-linked HA fillers might be expected better clinical outcomes, currently available scientific evidence did not confirm that hypothesis.

The location of this HA will critically depend on its concentration and the clinical effect that we are looking for; indeed, more concentrated HAs should be placed in the deeper areas of the skin and supraperiostal areas, while those with a lower concentration require a more superficial injection [63].

VYCROSS^®^ products’ formulation has a combination of low and high molecular weight. From the clinical point of view, the high molecular weight smoothes lines, furrows and wrinkles in the skin, while low molecular weight chains provide elasticity and structural support to it.

Regarding laser and intense light systems, there are two important concepts to understand the action of these devices on the skin, namely penetration and absorption.

Penetration refers to the capability of light to pass through a tissue, causing changes in it or not. The longer the wavelength, the greater the penetration, whereas absorption refers to the capacity of a tissue to trap light energy causing changes in it [46].

HA presents high absorption from lights with wavelengths of more than 1000 nm (nm), since the molar extinction coefficient of the HA for these wavelengths increases proportionally. The pulses or emission times used by the currently available laser and intense light systems refer to the time of light emission. These can be measured in seconds (s), milliseconds (ms), microseconds (s), nanoseconds (ns), and picoseconds (ps). The longer the pulse, the greater the penetration of light into the tissues [46].

Therefore, the interaction of the light in the tissues will depend on the electronic characteristics of the light, either its wavelength or pulse duration or the tissue light absorption, depending on the different coefficients of light molar extinction for the different chromophores (water, hemoglobin or melanin).

### Can Fillers be Successfully and Safely Used with Lasers, IPL, or Radiofrequency?

With the rising popularity of fractional laser treatments and soft tissue fillers, the interaction between laser/light treatments and soft tissue fillers is an area that is generating a considerable interest.

Both procedures aim to improve facial skin contour and rhytids using significantly different approaches. Anecdotal reports allege that the use of laser/light/RF devices after injection of filling substances might substantially reduce the effect of the fillers and/or lead to rapid degradation of the filling substances [64]. This is the reason it became common practice that when both HA filler implantation and laser therapy are used in the same patient, most specialists administer the laser therapy either before or after HA filler injection.

Although nonablative laser/light and superficial ablative treatments do not penetrate nearly deep enough to affect any fillers and can be safely used in combination, it is recommended to use the energy devices first [13].

However, the effect of common laser treatments over skin that has been injected with HA fillers has not been clearly elucidated in the literature.

A review of the literature published in 2015 identified seven studies involving combined light system treatments with fillers [65]. According to this review, six studies documented no histological changes in fillers injected after applying radiofrequency, IPL, or laser treatments and one studied documented improvement in collagen after IPL treatment and toxin injection [65].

The first study that evaluated the effects of monopolar radiofrequency treatment over soft tissue fillers was published by England et al. in 2005 [66]. This study examined, in a juvenile pig model, the tissue interactions of monopolar RF heating with five commonly injected fillers, namely cross-linked human collagen, HA, calcium hydroxylapatite, polylactic acid, and liquid injectable silicone [66]. The results found that there was no apparent increase in the risk of local burns and no observable effect of RF treatment on filler persistence in the tissue [66]. Moreover, filler presence did not increase the risk of undesirable thermal effects with monopolar RF treatment [66].

However, a second study performed by the same group found that although no immediate thermal effect of RF treatment was observed histologically, RF treatment resulted in statistically significant increases in the inflammatory, foreign body, and fibrotic responses associated with the filler substances [67].

The safety of RF treatment over skin areas recently injected with medium-term injectable soft tissue augmentation materials was assessed, in humans, by Alam et al. in 2006 [18]. Each subject received injections of 0.3 mL of hyaluronic acid derivative and calcium hydroxylapatite. Two weeks later, two nonoverlapping passes of RF were delivered over injected sites in all of the experimental subjects [18]. Based on the results of this study, to apply RF treatment over the same area 2 weeks after deep dermal injection with HA fillers or calcium hydroxylapatite does not appear to cause gross morphological changes in the filler material or surrounding skin [18].

Kim et al. [17] examined the clinical and histologic effects of a new needle that incorporates an RF device for HA injections. This study included three healthy Korean male volunteers all of whom were assessed to have nasolabial wrinkles rated as 2 (mild) or 3 (moderate) on the Wrinkle Severity Rating Scale (WSRS) [17]. All subjects were treated with RF on the right nasolabial fold before the filler injection, whereas the left side was treated with HA filler alone. The results of this study found that, concerning the change in WSRS scores at all post-baseline time points, subjects pretreated with RF achieved better outcomes than those treated with filler injections alone [17]. The procedure was well tolerated by all participants, none of whom reported any serious adverse events [17].

Similar results were reported by Choi et al. in ten Korean female volunteers with mild-to-severe nasolabial fold treated with a combination therapy of intradermal RF and HA filler [68]. This study found that intradermal RF treatment prior to HA filler injection may provide synergistic and long-lasting effects for the reduction in nasolabial fold wrinkles [68].

The effect of laser/light treatments on HA fillers [Restylane^®^ (Medicis, Scottsdale, AZ), Perlane^®^ (Medicis), and Juvéderm^®^ (Allergan, Irvine, CA, USA)] was evaluated in a porcine model [69]. Two weeks after injection, the injection sites were treated with 1 of 7 common laser/light ablative or nonablative devices [69]. This study concluded that, independently of the type of HA filler, following laser/light treatments, there was no sign of abnormal tissue damage or injury, or alteration of the filler, grossly or histologically, in the preinjected sites [69]. Caution is needed when planning superficial filler placement with aggressive deep laser/light technologies; in such a case, it is recommended to start with the laser treatment [69].

However, in a recently published study, which evaluated histologic changes, in abdominoplasty skin samples, after fractional laser and RF therapies, applied over preinjected HA fillers in the mid-to-deep dermis, we found that, although treatment with 1540-, 1550-, 1927-, and 10,600-nm lasers did not result in any morphologic changes of HA fillers, the RF devices demonstrated thermal damage of HA fillers along the microneedle tracks [70]. Therefore, caution is advised in using microneedle RF over recently injected HA. However, it should be noted that the study was not performed on facial skin [70].

Goldman et al., in a prospective, randomized, and evaluator-blind study, evaluated whether 1320-nm Nd:YAG laser, 1450-nm diode laser, monopolar RF, and/or IPL therapies could be safely administered immediately after HA gel treatment without compromising the effect of the dermal filler [15]. This study included 36 subjects, with prominent nasolabial folds, who were treated with HA gel implantation on one side of the face and hyaluronic acid gel followed by one of the nonablative laser/RF/IPL therapies on the contralateral side of the face [15]. The results of this study found that laser, RF, and IPL treatments can safely be administered immediately after hyaluronic acid gel implantation without reduction in overall clinical effect [15].

The interaction between a HA filler followed immediately by laser was assessed in nine women that underwent neck-skin rejuvenation [71]. The results of the study indicated improvements in fine wrinkles, tightness, and skin texture. Additionally, histologic evaluations showed favorable changes in cellularity, collagen, and elastic fibers. The laser-induced effects and an inflammatory reaction were seen at 400 and 1000 μm, respectively, whereas the HA filler was present at the mid-deep dermis (1000–1500 μm) [71].

Park et al. [14] conducted a study that evaluated the potential for synergistic effects with combined treatment using a nonablative infrared device and HA filler in the treatment of nasolabial fold wrinkles. According to the results of this study, combining the use of a nonablative infrared device with HA filler does not appear to be superior to HA filler alone in the treatment of moderate-to-severe nasolabial fold wrinkles [14].

Table 2 summarizes the capacity of different wavelengths to be safely used with different dermal fillers.

**Table 2:** Capacity of different wavelengths to be safely used with different dermal fillers

Fillers	Wavelengths										
	IPL’S (< 950 nm)	532 nm Q-S	650 nm	694 nm	755 nm	810 nm	1064 nm Q-S	1064 nm	1450 nm	1550 nm	2940 nm
ULTRA 2	Y	Y	Y	Y	Y	Y	Y	N	N	N	N
ULTRA 3	Y	Y	Y	Y	Y	Y	Y	N	N	N	N
ULTRA 4	Y	Y	Y	Y	Y	Y	Y	N	N	N	N
VOLUMA LIDO	Y	Y	Y	Y	Y	Y	Y	N	N	N	N
VOLIFT	Y	Y	Y	Y	Y	Y	Y	N	N	N	N
VOLVELLA	Y	Y	Y	Y	Y	Y	Y	N	N	N	N
VOLITE	Y	Y	Y	Y	Y	Y	Y	N	N	N	N

*IPL* intense pulse light,*Y* yes, *N* no

## Conclusions

The currently available scientific evidence about the combined use of HA fillers and laser/RF/IPL includes small and nonrandomized studies. Nevertheless, most of these studies found that, on average, the concomitant use (same day) of laser and HA fillers for facial rejuvenation represents an effective and safe strategy which improve clinical results and patient’s satisfaction.

This consensus report was focused on the HA fillers Juvederm VYCROSS^®^ (Allergan, Irvine, CA, USA) at different concentrations, namely 12.5 mg; 15 mg; 17.5 mg and 20 mg. Nevertheless, all of them have extremely low and constant levels of 1,4-butanediol diglycidyl ether (BDDE).

The difference in timing (waiting 1 or 2 h) between laser treatment and HA filler injection is not decisive; what really matters is the sequence of the treatments (laser first and subsequently HA injection) and the wavelength of the laser.

As the limitation of this consensus, it should be mentioned that all the discussion and the recommendations circumscribed to the branded Allergan HA fillers (Allergan, Irvine, CA, USA).

The panel recommendations are:

- If we want to perform a combined procedure on the same day (HA and light treatments), start always with the light treatments, avoiding skin manipulations after having injected HA.
- In the aforementioned procedure, light systems will be always nonablative, minimizing the risk of wounds in the skin that can cause infections.
- In the retreatment light sessions, after treatments with HA, we will avoid the use of lights or lasers with wavelengths higher than 1000 nm, with a pulse duration of milliseconds, especially when we have previously used HA in supraperiostal localization or superficial or medium dermal injections. As far as we know, there have not been any problems or interactions with other nonablative lasers of lower wavelengths.
- In the retreatment sessions, all light systems, which use pulse durations in microseconds, nanoseconds, or picoseconds, regardless of the wavelength used, may be used after any HA.
- The depth of the injected filler is another important aspect to take into account when performing a combined procedure on the same day (HA and light treatments). The different HA fillers are injected at different depths, ranging from supraperiosteal location to middle papillary dermis. That is the reason for recommending the use of nonablative lasers (any wavelength and any pulse duration) and later on, without fixed time, proceeding to the AH filler injection. A prospective study evaluating the elapsed time between laser and HA filler, as well as the impact of HA filler concentration and depth of injections, may give better understanding of the outcomes.
- A correct diagnosis of the photodamage and loss of volumes suffered by the patients will help us to choose and properly tailor our therapeutic management, combining properly photodamage and loss of volume treatments in the same session.
- Although both strategies are relatively safe, they are not exempt from the appearance of possible complications. Most of the complications are transient in nature and can be successfully treated. The panel considers that an adequate selection of the patient, technique and filler will help to ensure a desirable outcome.

Future well-designed clinical studies are needed regarding the effectiveness and safety of combined filler/laser treatments.
